# Protective Immunity to *Mycobacterium tuberculosis* Infection by Chemokine and Cytokine Conditioned CFP-10 Differentiated Dendritic Cells

**DOI:** 10.1371/journal.pone.0002869

**Published:** 2008-08-06

**Authors:** Nasir Salam, Shashank Gupta, Sachin Sharma, Shweta Pahujani, Aprajita Sinha, Rajiv K. Saxena, Krishnamurthy Natarajan

**Affiliations:** 1 Immunology Group, International Centre for Genetic Engineering and Biotechnology, Aruna Asaf Ali Marg, New Delhi, India; 2 School of Life Sciences, Jawaharlal Nehru University, New Delhi, India; Harvard Medical School, United States of America

## Abstract

**Background:**

Dendritic cells (DCs) play major roles in mediating immune responses to mycobacteria. A crucial aspect of this is the priming of T cells via chemokines and cytokines. In this study we investigated the roles of chemokines RANTES and IP-10 in regulating protective responses from *Mycobacterium tuberculosis* (*M. tb*) 10 kDa Culture Filtrate Protein-10 (CFP-10) differentiated DCs (CFP10-DCs).

**Methods and Findings:**

Infection of CFP10-DCs with mycobacteria down-modulated RANTES and IP-10 levels. Pathway specific microarray analyses showed that in addition to RANTES and IP-10, mycobacteria infected CFP10-DCs showed reduced expression of many Th1 promoting chemokines and chemokine receptors. Importantly, T cells co-cultured with RANTES and IP-10 conditioned CFP10-DCs mediated killing of mycobacteria from infected macrophages. Similarly, T cells recruited by RANTES and IP-10 conditioned CFP10-DCs mediated significant killing of mycobacteria from infected macrophages. IFN-gamma treatment of CFP10-DCs restored RANTES and IP-10 levels and T cells activated by these DCs mediated significant killing of virulent *M. tb* inside macrophages. Adoptive transfer of either RANTES and IP-10 or IL-12 and IFN-gamma conditioned CFP10-DCs cleared an established *M. tb* infection in mice. The extent of clearance was similar to that obtained with drug treatment.

**Conclusions:**

These results indicate that chemokine and cytokine secretion by DCs differentiated by *M. tb* antigens such as CFP-10 play major roles in regulating protective immune responses at sites of infection.

## Introduction

*Mycobacterium tuberculosis* (*M. tb*) continues to cause over 2 million deaths annually. This problem is further complicated by the emergence of multi-drug resistant strains and the variable efficiency of protection offered by vaccination with *M. bovis* BCG (hereafter BCG) [1]. Therefore, this underscores the need to elucidate factors that regulate protective immune responses against this pathogen. Among the antigen presenting cells of the immune system, Dendritic Cells (DCs) play critical roles in initiating protective responses to pathogens [2] and act as a bridge between the innate and the acquired arm of the immune system. This is largely attributed to their ability to stimulate naïve quiescent T cells and thereby initiate a primary immune response. Following sensing of infection, DCs are recruited to sites of infection, where they take-up antigens/pathogens and initiate T cell responses. Depending upon the activation status, DCs initiate either inflammatory or regulatory responses that determine clearance of infection [3].

Although macrophages are the preferred hosts for mycobacteria, *M. tb* infects DCs as well that are crucial to prime T cells and regulate mycobacterial survival in the host [4]. Among the factors that regulate protective immunity are cytokines and chemokines [5]. Cytokines such as Interferon (IFN)-γ and Interleukin (IL)-12 are known to offer protective immune responses to *M. tb* [6]. Likewise chemokines such as Regulated upon Activation Normal T cell Expressed and Secreted (RANTES) and Interferon-Inducible Protein (IP)-10 play major roles in mediating protective responses in addition to mediating chemotaxis [6].

Previously we showed that 10 kDa *M. tb* Culture Filtrate Protein-10 (CFP-10, also known as MTSA-10) and many other *M. tb* antigens like ESAT-6, Ag85B and MPT64 induce the differentiation and maturation of DCs [7], [8]. DCs differentiated with CFP-10 (hereafter CFP10-DCs) are very similar to DCs differentiated conventionally with GM-CSF (hereafter GM-CSF-DCs), in terms of phenotype, morphology and maturation status based on the density and profile of surface markers [7]. The two DCs express similar levels of costimulatory and MHC molecules and are essentially immature. However, a challenge of CFP10-DCs with mycobacterial extract downregulates the expression of IL-12p40 and T cells co-cultured with these DCs induce suppressor responses with high levels of IL-10 and low levels of IL-2 and IFN-γ [8], [9]. In contrast, a similar challenge of GM-CSF-DCs upregulates IL-12p40 and co-culture with T cells induces pro-inflammatory responses with high levels of IFN-γ and low levels of IL-10. These results indicate that while CFP10-DCs and GM-CSF-DCs share phenotypic similarities; their functional responses are quite different. Further, these results also indicate that DC-differentiation by antigens such as CFP-10 could be a strategy by mycobacteria to induce suppressor responses.

In order to characterize the mechanisms by which CFP10-DCs induce suppressor responses, in this study, we examined the roles played by chemokines RANTES and IP-10 in regulating pro-inflammatory responses from CFP10-DCs. We report that challenging CFP10-DCs with live BCG downregulates the expression of RANTES and IP-10 and many other chemokines that induce pro-inflammatory responses. Conditioning CFP10-DCs with RANTES or IP-10 induced Th1 responses. Importantly, conditioning CFP10-DCs with either RANTES and IP-10 or IFN-γ and IL-12 mediates effective clearance of an established *M. tb* infection in mice, thereby giving functional relevance to the above observations.

## Results

### Stimulation of CFP10-DCs with BCG downregulates RANTES and IP-10 levels

**Figure 1:**
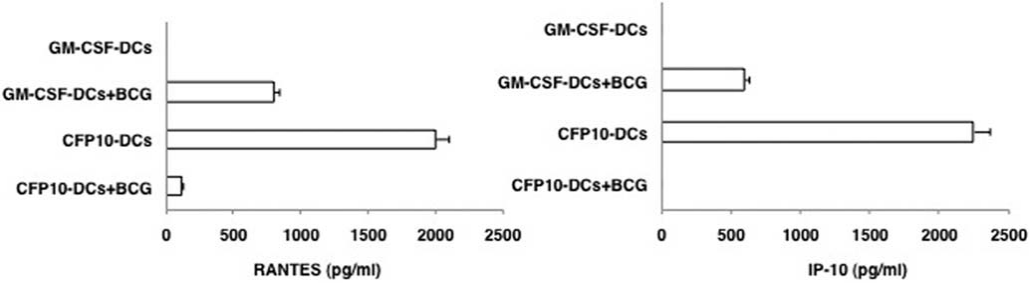
CFP10-DCs downregulate RANTES and IP-10 levels following BCG infection. GM-CSF-DCs or CFP10-DCs were infected with 1 MOI BCG for 24h. RANTES and IP-10 levels in culture supernatants were measured by ELISA. Data from one of five experiments is shown.

To begin with we investigated the levels of RANTES and IP-10 in CFP10-DCs and GM-CSF-DCs upon BCG infection. Compared to unstimulated GM-CSF-DCs, unstimulated CFP10-DCs secreted high levels of both RANTES and IP-10 (Figure 1). This indicated that secretion of RANTES from DCs requires a microbial stimulus [6] that was provided by CFP-10 during DC-differentiation. BCG stimulation of GM-CSF-DCs boosted the levels of RANTES and IP-10 by 500-fold. On the other hand BCG stimulated CFP10-DCs showed a 95% decrease in RANTES, while IP-10 levels were undetectable, when compared with uninfected CFP10-DCs. These results indicated that CFP10-DCs downregulate expression of pro-inflammatory chemokines following their interactions with live mycobacteria. We confirmed that BCG infection induced a similar level of maturation of GM-CSF-DCs and CFP10-DCs based on the increase in the surface densities of a number of markers that are reflective of mature DCs (Figure S1, panel A). In addition, the two DCs showed a similar uptake of labeled BCG [10] (Figure S1, panel B). This indicated that lower production of RANTES and IP-10 by BCG infected CFP10-DCs did not result from reduced bacterial uptake. Further, to rule out the possibility that downregulation of RANTES and IP-10 by CFP10-DCs following BCG infection is not because the DCs were refractory to additional stimulation, we treated both GM-CSF-DCs and CFP10-DCs with LPS and looked at the levels of RANTES and IP-10. As shown in Figure S2, LPS addition of both GM-CSF-DCs and CFP10-DCs upregulated the levels of both RANTES and IP-10, indicating that LPS induced activation of both DCs. Importantly, this also indicated that CFP10-DCs were not refractory to additional stimulation. Further, this also suggested that CFP10-DCs downregulate RANTES and IP-10 expression specifically following mycobacterial stimulation.

CFP-10 has been shown to dimerize with Early Secretory Antigenic Target (ESAT6) and immune responses induced by CFP-10:ESAT6 dimer have been shown to be similar to that induced by CFP-10 monomer [11], [12]. We, therefore, generated CFP10:ESAT6 dimer (Figure S3) and monitored RANTES and IP-10 levels from DCs differentiated by CFP10:ESAT6 dimer (Dimer-DCs). Like CFP10-DCs, Dimer-DCs secreted high levels of RANTES and IP-10. However, stimulation of Dimer-DCs with BCG, significantly downregulated RANTES and IP-10 levels (Figure S4).

It has been reported that the transcription factor NF-κB binding site at position -67 to -99 in the mouse RANTES promoter is critical for transcription and expression of RANTES [13]. Likewise, the NF-κB site in IP-10 promoter at position -99 to -118 is crucial for IP-10 expression [14]. Therefore, we investigated the recruitment of NF-κB to their respective promoters by EMSA. BCG infection of GM-CSF-DCs recruited significantly higher levels of NF-κB to both RANTES and IP-10 promoters (Figure S5). These results indicate that reduced levels of RANTES and IP-10 observed in BCG infected CFP10-DCs could result from reduced NF-κB recruitment to their promoters.

### Conditioning CFP10-DCs with RANTES or IP-10 induces primary pro-inflammatory responses to BCG

**Figure 2:**
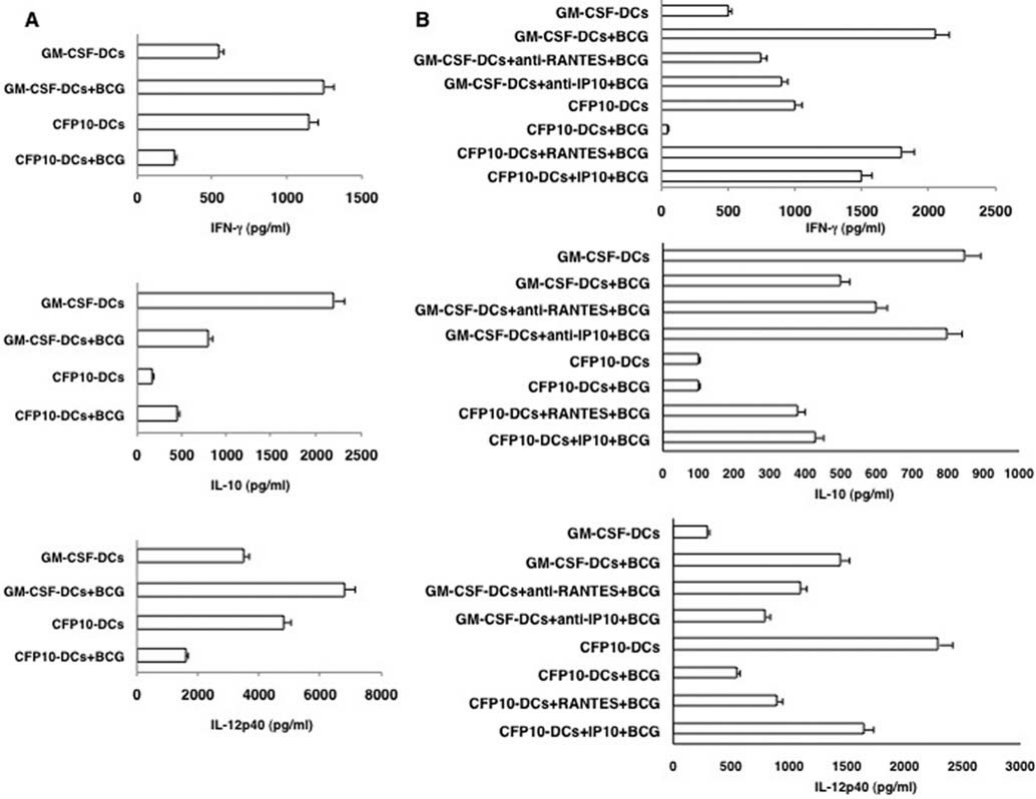
RANTES and IP-10 conditioned CFP10-DCs induce pro-inflammatory in vivo primary immune responses to BCG. For Panel A GM-CSF-DCs or CFP10-DCs were infected in vitro with 1 MOI BCG for 24h. DCs were extensively washed to remove extracellular bacteria. 5×10^6^ DCs were adoptively transferred into naïve mice. Seven days later the inguinal lymph nodes were removed and 5×10^6^ cells/ml were cultured in 10% RPMI1640 medium with 10% FCS for 48h. Levels of indicated cytokines were measured in culture supernatants. For B, prior to infection with BCG, GM-CSF-DCs were either untreated or incubated with neutralizing monoclonal antibody to RANTES or IP-10 for 4h, while CFP10-DCs were either untreated or incubated with 25 ng/ml RANTES or IP-10 for 12h. DCs were extensively washed and then processed as in Panel A. Data from one of three experiments are shown.

To give functional implications to the data obtained in Figure 1, we investigated if CFP10-DCs induce suppressor in vivo primary responses to BCG and if conditioning DCs with RANTES or IP-10 could induce pro-inflammatory responses. As shown in Figure 2A, uninfected CFP10-DCs gave pro-inflammatory responses with high levels of IFN-γ and IL-12p40 and low levels of IL-10. However, BCG infected CFP10-DCs resulted in a complete reversal of pro-inflammatory responses into suppressor responses with a ∼5-fold downregulation of IFN-γ levels and ∼2-fold downregulation of IL-12p40 levels, along with a marginal increase in IL-10 levels. In contrast, BCG infected GM-CSF-DCs showed a classical pro-inflammatory response with >2-fold increase in levels of IFN-γ and a ∼2-fold increase in IL-12p40 levels. These results indicated that CFP10-DCs induce suppressor primary responses to live mycobacteria.

Next, we investigated if priming CFP10-DCs with RANTES or IP-10 would induce pro-inflammatory responses to BCG. To this end CFP10-DCs were conditioned with either RANTES or IP-10 prior to infection with BCG. Likewise, GM-CSF-DCs were treated with neutralizing antibodies to RANTES or IP-10 prior to BCG infection. As shown in Figure 2B, incubating GM-CSF-DCs with neutralizing antibodies to either RANTES or IP-10 reduced IFN-γ levels by ∼50% when compared in the absence of neutralization. IL-12p40 levels were also reduced, more significantly upon neutralizing IP-10. Incubation with isotype matched non-specific antibodies had no effect (data not shown). In contrast, conditioning CFP10-DCs with either RANTES or IP-10 now induced pro-inflammatory responses with a 10-fold increase in IFN-γ levels and a 2-fold increase in IL-12p40 levels when compared in the absence of any treatment.

The results in Figure 2 thus indicate that downregulated levels of RANTES and IP-10 in CFP10-DCs play a dominant role in regulating the generation of pro-inflammatory immune responses to mycobacteria. Further, these results are consistent with the ability of RANTES and IP-10 to induce pro-inflammatory responses from DCs in response to a microbial infection [15].

### RANTES and IP-10 conditioned CFP10-DCs increase calcium influx

**Figure 3:**
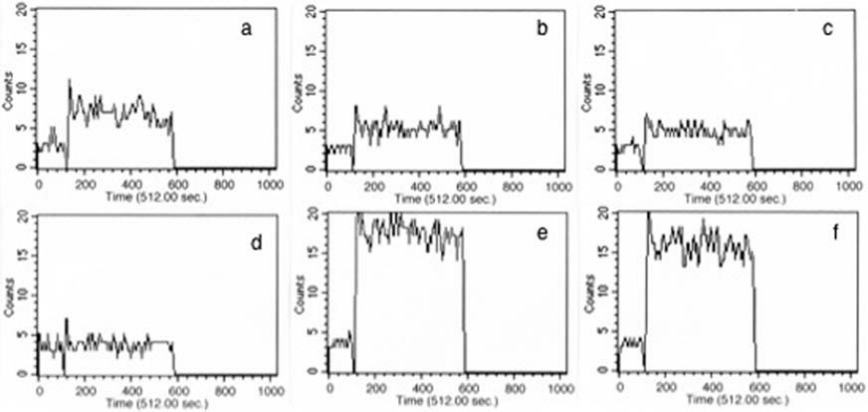
RANTES and IP-10 elevate intracellular calcium in BCG stimulated CFP10-DCs. FLUO-3-AM labeled GM-CSF-DCs (profile a–c) or CFP10-DCs (profile d–f) were stimulated with 1 MOI BCG and real-time increase in intracellular calcium was monitored over a period of 5 min. For profiles b and c GM-CSF-DCs were incubated with 5 μg/ml neutralizing monoclonal antibody to RANTES and IP-10, respectively, for 2h, prior to BCG stimulation. For profiles e and f, CFP10-DCs were incubated with 5 ng/ml recombinant RANTES and IP-10, respectively, for 12h, prior to BCG stimulation. DCs were extensively washed prior to flow cytometry. Data from one of three experiments are shown.

Stimulation of DCs with a microbial stimulus is known to mobilize intracellular calcium that favors pro-inflammatory Th1 responses [16]. We have also shown earlier that calcium influx in CFP10-DCs is compromised that results in increased survival of mycobacteria in CFP10-DCs [17]. It was thus of interest to next investigate if RANTES and IP-10 would modulate calcium influx. As shown in Figure 3, stimulation of GM-CSF-DCs with BCG induced higher (392 nM) intracellular calcium (Figure 3 profile a) as compared to CFP10-DCs that induced a much lower (83 nM) calcium influx (Figure 3 profile d), reiterating that calcium responses are suppressed in CFP10-DCs. However, treatment of GM-CSF-DCs with neutralizing antibodies to either RANTES or IP-10 (Figure 3 profiles b and c) significantly attenuated the increase in intracellular calcium. This indicated that RANTES and IP-10 play direct roles in calcium mobilization in DCs following mycobacterial challenge. Further, neutralizing either RANTES or IP-10 alone in GM-CSF-DCs attenuated calcium influx only partially. This indicated that both chemokines are equally important in influencing calcium influx in DCs following mycobacterial stimulation and could play individual and cooperative roles to this end. These results correlate well with the results in Figure 2 wherein a reduced but not completely attenuated levels of IFN-γ and IL-12p40 levels were obtained upon neutralizing either RANTES or IP-10 in BCG infected GM-CSF-DCs. Conversely, CFP10-DCs treated with either RANTES or IP-10 (Figure 3 profiles e and f) readily increased intracellular calcium concentration up to 835 nM and 876 nM, respectively, upon BCG stimulation. These results suggested that RANTES and IP-10, conditioned CFP10-DCs to be responsive to increase in intracellular calcium following stimulation by BCG. Further, this also suggested that increasing intracellular calcium levels could be a possible mechanism of induction of pro-inflammatory responses from CFP10-DCs by the two chemokines.

### BCG infected CFP10-DCs express reduced levels of genes promoting pro-inflammatory responses

**Figure 4:**
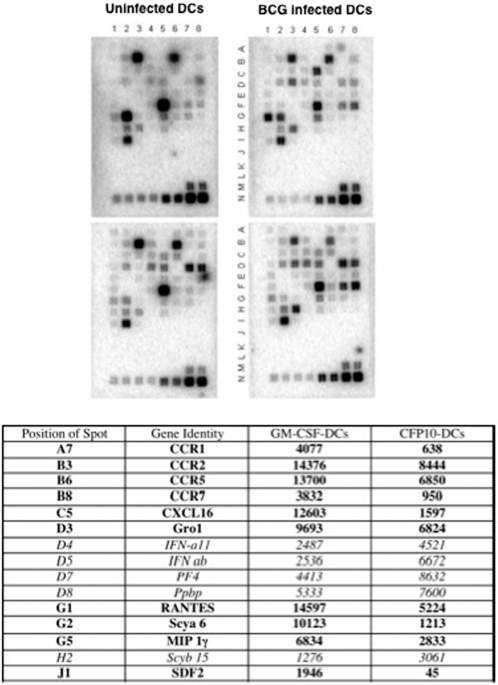
BCG infected CFP10-DCs show reduced expression of pro-inflammatory chemokines. Total RNA was enriched from either GM-CSF-DCs (upper panels) or CFP10-DCs (lower panels) following infection with 1 MOI BCG (upper right and lower right panels) for 24h. 1 μg RNA was processed for microarray analyses using the GEArray Q Series mouse chemokine and receptor gene array from SuperArray strictly following the manufacturer's instructions. Rows A–I contains genes for various chemokines and their receptors while rows M and N contain house-keeping genes. Rows J–L represents negative controls. The Table below the figure depicts genes, their position and densitometric units of spots from blots of BCG infected GM-CSF-DCs and BCG infected CFP10-DCs. Data from one of two experiments are shown.

We next applied a pathway specific microarray approach to investigate if stimulation of GM-CSF-DCs and CFP10-DCs with BCG would differentially alter the expression profiles of other chemokines and their receptors. As shown in Figure 4, the expression patterns of most genes were more or less similar in uninfected GM-CSF-DCs and CFP10-DCs. Some differences were however observed. For example, the expression of Cmbkr1/1(spot # B2), IFN-a11(spot # D4), IFNab(spot # D5), PF4(spot # D7) and Ppbp(spot # D8) were higher in CFP10-DCs as compared to GM-CSF-DCs. Interestingly, however, compared to protein levels, message levels of RANTES in uninfected CFP10-DCs and GM-CSF-DCs were similar. This could be attributed to the fact that CFP10-DCs secrete high levels of RANTES even during the DC-differentiation process (data not shown). Since the message levels were analyzed in fully differentiated DCs, this could be due to a feedback regulation at the transcript level or differences in the half-life and/or the translational rates in the two DCs.

Importantly, infection with BCG displayed significant differences in the expression of a number of chemokines and their receptors. Many genes were weakly expressed, while the expression of many genes [Ppbp(spot # D8), MIP 1a(spot # F7) and MIP-1(spot # H3)] was nearly equal. Significant differences in the expression levels of 15 genes were however observed. As seen from the Table appended under Figure 4, based on the densitometric values the expression levels of 10 genes were higher in BCG infected GM-CSF-DCs (represented as **bold**), while the expression levels of 5 genes were higher in BCG infected CFP10-DCs (represented as *italics*). The expression of genes that favor pro-inflammatory responses such as RANTES, Gro1, SDF-1, MIP-1γ, CXCL16 [15] showed higher expression in BCG infected GM-CSF-DCs when compared with BCG infected CFP10-DCs. Interestingly, however, IP-10 message levels were not detectable in any group perhaps reflecting a tight regulation at the transcriptional or post-transcriptional levels. The chemokine CXCL16 plays a very important role in the recruitment of activated effector and memory CD4^+^ T cells [18], [19]. In addition, the expression levels of a number of chemokine receptors such as CCR1, CCR2, CCR5 and CCR7 was lower in BCG infected CFP10-DCs when compared with BCG infected GM-CSF-DCs. The increased expression of these receptors e.g. CCR5 may increase the avidity of the interaction of RANTES with CCR5 and/or CCR1 (another receptor for RANTES). Increased expression of CCR7 might also aid in faster migration of bacteria containing GM-CSF-DCs to T cell areas and mediate T cell priming.

The expression levels of Thymocyte Activated and Regulated Chemokine (TARC)(spot # E5) and Eotaxin(spot # E3) that promote suppressor responses [6], were also weakly increased in both uninfected and BCG infected CFP10-DCs, while their expression was below detectable levels in GM-CSF-DCs.

More significantly the expression levels of type 1 interferons (IFNs)-IFN ab and IFN a11- were highly expressed in uninfected CFP10-DCs and increased further following BCG infection. It has been reported that exposure of DCs to IFN-β prior to T cell engagement and during CD40-CD40L cross-talk inhibits Th1 cell polarization and promotes the generation of IL-10 producing T cell subsets [20], [21]. Thus, BCG infection of GM-CSF-DCs induces the selective expression of pro-inflammatory and T cell recruiting chemokines, along with increase in the expression of their receptors that might increase the avidity of the interaction. In contrast, BCG infected CFP10-DCs increase the expression of chemokines that favor suppressor responses.

### T cells cultured with RANTES and IP-10 conditioned BCG infected CFP10-DCs mediate enhanced killing of mycobacteria

**Figure 5:**
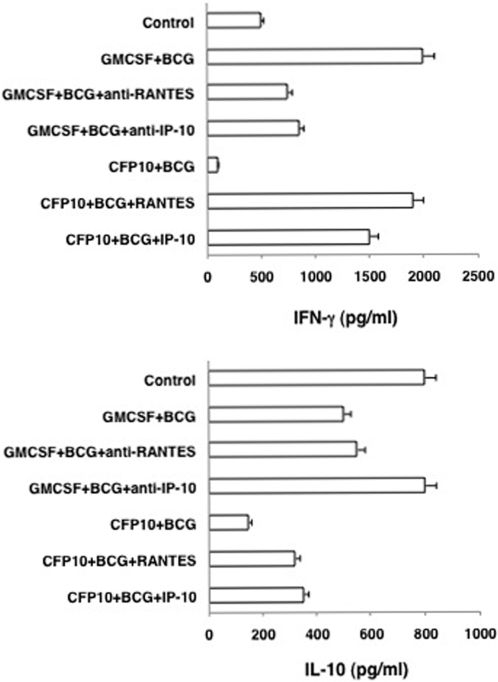
RANTES and IP-10 conditioned CFP10-DCs induce Th1 responses. Either GM-CSF-DCs (GMCSF) or CFP10-DCs (CFP10) were infected with 1 MOI BCG for 24h and co-cultured for 48h with T cells enriched from BCG immunized mice. For some groups GM-CSF-DCs were incubated with 5 μg/ml neutralizing monoclonal antibody to RANTES and IP-10, respectively, for 2h, prior to BCG stimulation. CFP10-DCs were incubated with 5 ng/ml recombinant RANTES or IP-10 for 12h, prior to BCG stimulation. Data from one of three independent experiments are shown.

An important function of DCs is to prime T cells that subsequently activate infected macrophages to kill/restrict intracellular pathogens including *M. tb* [22]. To this end we first investigated whether T cells co-cultured with RANTES or IP-10 conditioned BCG infected CFP10-DCs have a Th1 phenotype. As shown in Figure 5, T cells from BCG immunized mice co-cultured with BCG infected GM-CSF-DCs secreted high levels of IFN-γ as compared to IL-10, whereas T cells from BCG immunized mice co-cultured with BCG infected CFP10-DCs displayed a Th0 phenotype with low levels of IFN-γ and IL-10. However, neutralizing either RANTES or IP-10 in GM-CSF-DCs reduced IFN-γ secretion by 4-fold. On the other hand, treating CFP10-DCs with either RANTES or IP-10 induced a characteristic Th1 response with high levels of IFN-γ as compared to IL-10. These results indicated that conditioning CFP10-DCs with RANTES or IP-10 induced pro-inflammatory Th1 responses to BCG.

**Figure 6:**
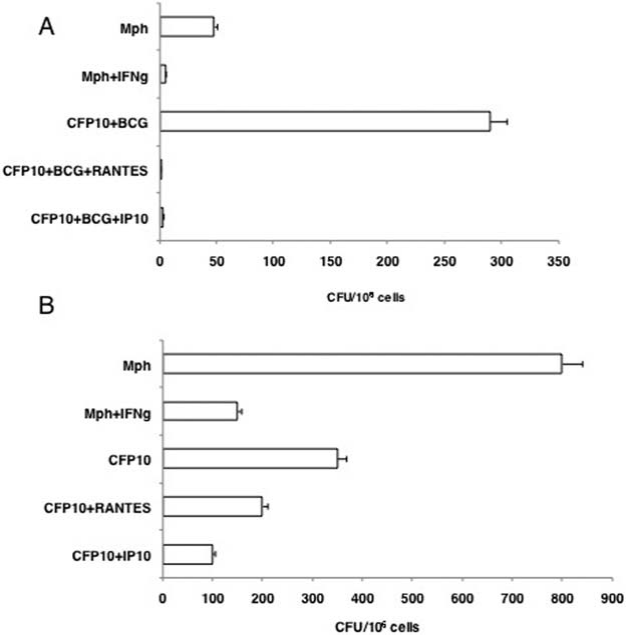
T cells co-cultured with or recruited by RANTES or IP-10 conditioned CFP10-DCs mediate clearance of BCG from macrophages. For A, BCG infected CFP10-DCs (CFP10), conditioned or not with either 25 ng/ml RANTES or IP-10 were co-cultured for 48h with BCG primed T cells. From this, T cells were enriched and cultured with BCG infected macrophages (Mph) for 48h. A separate group wherein macrophages conditioned with 2 ng/ml IFN-γ (IFN-g) for 4h prior to infection with BCG was also included as a control. For B, T cells migrated into the lower chamber of a transwell apparatus in response to supernatants of BCG infected CFP-DCs, conditioned or not with 25 ng/ml RANTES or IP-10, were cultured with BCG infected macrophages (Mph) for 48h. A separate group wherein macrophages conditioned with 2 ng/ml IFN-γ (IFN-g) for 4h prior to infection with BCG was also included as a control. Cells from both Panels were lysed and plated in serial dilutions on 7H11 agar plates. CFU were counted 2–3 week later. Data are the mean±s.d. of three experiments. For A, P<0.002 (Mph vs CFP10+BCG+RANTES), P<0.004 (Mph vs CFP10+BCG+IP10). For B, P<0.02 (Mph vs CFP10+BCG+RANTES), P<0.01 (Mph vs CFP10+BCG+IP10).

Next, in order to give functional relevance to the above observations we looked at the ability of T cells co-cultured with RANTES or IP-10 conditioned BCG infected CFP10-DCs in mediating killing of BCG inside macrophages. As shown in Figure 6A, T cells co-cultured with BCG infected CFP10-DCs displayed higher bacterial burden when compared to control (BCG infected macrophages), thereby inhibiting killing of BCG by macrophages. These results are concurrent with our earlier observations on increased bacterial loads in CFP10-DCs [17]. However, T cells co-cultured with BCG infected RANTES or IP-10 conditioned CFP10-DCs mediated effective killing of intracellular BCG in macrophages and reduced bacterial loads by 10-fold. In fact, the bacterial loads in these groups were similar to groups where macrophages were conditioned with recombinant IFN-γ prior to BCG infection, indicating that enhanced killing could be mediated by increased secretion of IFN-γ by T cells. This indicated that lower production of RANTES or IP-10 in mycobacteria infected CFP10-DCs contributed towards defective killing of intracellular mycobacteria by interacting T cells.

### T cells recruited by RANTES and IP-10 conditioned CFP10-DCs mediate effective killing of BCG

We extended the above observations to see if T cells recruited by RANTES and IP-10 would mediate killing of mycobacteria inside macrophages. We first investigated the activation status of T cells recruited by BCG infected CFP10-DCs and GM-CSF-DCs. BCG infected GM-CSF-DCs recruited a higher percentage of CD69^+^CD4^+^ T cells as compared with BCG infected CFP10-DCs (Figure S6). This indicated that CFP10-DCs are impaired in their ability to recruit activated T cells. These results correlated well with the results obtained in Figure 6A, wherein T cells co-cultured with BCG infected CFP10-DCs promoted higher bacterial burden in macrophages. Therefore, we next investigated whether, T cells recruited by supernatants of RANTES and IP-10 conditioned DCs would mediate effective killing of mycobacteria inside macrophages. As shown in Figure 6B T cells recruited by either RANTES or IP-10 conditioned BCG infected CFP10-DCs mediated significant reduction in the bacterial loads inside macrophages. These results indicated that RANTES and IP-10 play a direct role in the recruitment of effector T cells that have the ability to kill mycobacteria inside macrophages.

### RANTES and IP-10 conditioned CFP10-DCs mediate effective clearance of M. tb infection in mice

**Figure 7:**
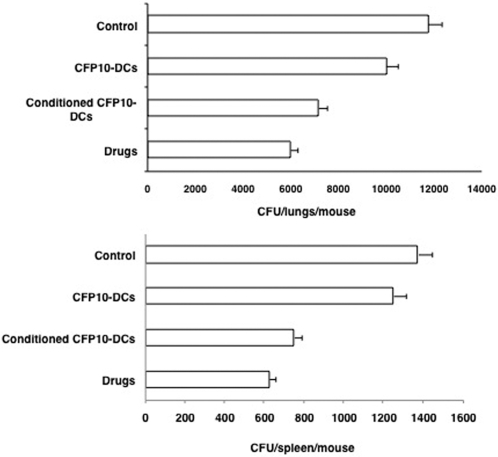
RANTES and IP-10 conditioned CFP10-DCs mediate clearance of *M. tb* infection in vivo in mice. Groups of mice were infected with 1×10^6^ *M. tb* H37Ra intravenously via the tail vein. Seven days post-infection, 10×10^6^ unconditioned CFP10-DCs or CFP10-DCs conditioned with both RANTES and IP-10 together (25 ng/ml each) for 12h (conditioned CFP10-DCs) were injected into the tail vein of mice. A repeat injection was given 7 days following the first transfer. Seven days following the 2^nd^ transfer, mice were sacrificed and lung and spleen homogenates were plated onto 7H11 agar plates in serial dilutions for CFU monitoring. In parallel, infected mice were injected with anti-TB drugs as given in *Materials and Methods*. Control represents mice infected with *M. tb* H37Ra followed by intravenous injection of PBS. Data are the mean±s.d. of three experiments. For lungs, P<0.02 (Control vs Conditioned CFP10-DCs), P<0.009 (CFP10-DCs vs conditioned CFP10-DCs). For spleen, P<0.006 (Control vs Conditioned CFP10-DCs), P<0.01 (CFP10-DCs vs conditioned CFP10-DCs).

We next investigated whether RANTES and IP-10 conditioned CFP10-DCs could provide protective immune responses to *M. tb* infection in vivo. To this end, we first established an infection with *M. tb* H37Ra in mice and then adoptively transferred CFP10-DCs conditioned with both RANTES and IP-10 together (in order to boost their effects) into infected mice. Bacterial burden in the lungs and spleen were subsequently monitored. In parallel, we also induced chemotherapy with drugs following established protocols (*see* *Materials and Methods*). As shown in Figure 7, bacterial burden in the lungs and spleen of mice that received CFP10-DCs were similar to the bacterial burden in control mice that received PBS alone, indicating that CFP10-DCs on their own are unable to clear *M. tb* infection. However, transfer of RANTES and IP-10 conditioned CFP10-DCs induced effective and significant reduction in the bacterial loads in both lungs and spleen. In fact, the extent of clearance was similar to that obtained with drug treatment indicating similar kinetics of clearance. These results indicate a direct role for RANTES and IP-10 in mediating clearance of an established *M. tb* infection.

### Treatment with IFN-γ rescues RANTES and IP-10 levels in BCG infected CFP10-DCs

**Figure 8:**
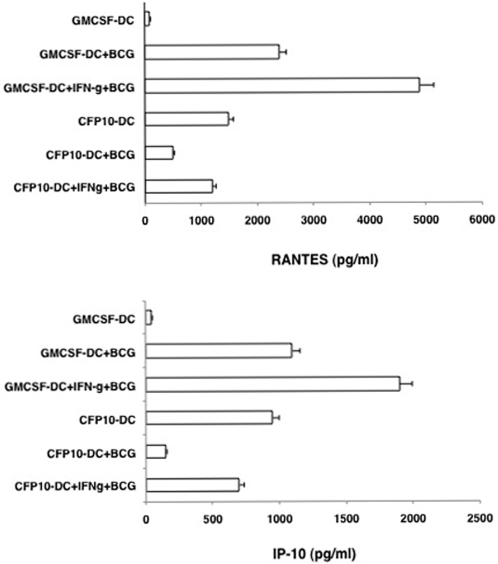
Treatment with IFN-γ restores RANTES and IP-10 levels from BCG infected CFP10-DCs. Either GM-CSF-DCs or CFP10-DCs were incubated with 2 ng/ml IFN-γ for 1h and washed. DCs were infected with 1 MOI BCG for 24h. Expression of RANTES and IP-10 was measured in culture supernatants. Data from one of three experiments are shown.

We had earlier shown that conditioning CFP10-DCs with IFN-γ induced Th1 responses to mycobacteria [9]. We, therefore, investigated if IFN-γ could also reverse the decrease in the levels of RANTES and IP-10 in CFP10-DCs following BCG infection. As shown in Figure 8, treatment of CFP10-DCs with IFN-γ increased RANTES and IP-10 levels from CFP10-DCs following BCG infection. Expectedly, treatment of GM-CSF-DCs with IFN-γ further enhanced the levels of the two chemokines.

### IFN-γ and IL-12 conditioned CFP10-DCs clear M. tb infection in mice

**Figure 9:**
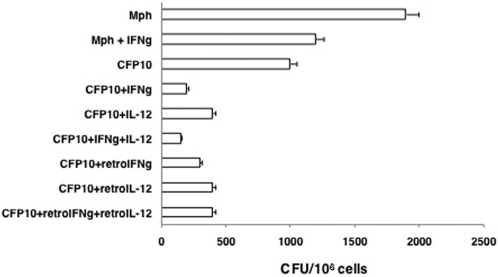
T cells co-cultured with IFN-γ or IL-12 conditioned CFP10-DCs mediate clearance of *M. tb* H37Rv from macrophages. BCG infected CFP10-DCs (CFP10), conditioned or not with either 2 ng/ml IFN-γ (IFNg) or IL-12 or transformed with retroviruses expressing IFN-γ (retroIFNg) or IL-12p40 (retroIL-12), were co-cultured for 48h with BCG primed T cells. From this, T cells were enriched and cultured with *M. tb* H37Rv infected macrophages (Mph) for 48h. A separate group wherein macrophages conditioned with 2 ng/ml IFN-γ, prior to infection with *M. tb* H37Rv was also included as a control. Cells from all the groups were lysed and plated in serial dilutions on 7H11 agar plates for CFU monitoring 2–3 week later. Data are the mean±s.d. of three experiments. P<0.007 (Mph vs CFP10+IFNg), P<0.007 (Mph vs CFP10+IL-12); P<0.005 (Mph+IFNg vs CFP10+IFNg), P<0.005 (Mph+IFNg vs CFP10+IL-12); P<0.03 (CFP10 vs CFP10+IFNg), P<0.05 (CFP10 vs CFP10+IL-12).

We next investigated whether, similar to RANTES and IP-10, conditioning CFP10-DCs with IFN-γ and/or IL-12 would offer protective immunity to *M. tb* infection both in vitro and in vivo. We first investigated whether, T cells activated by IFN-γ and IL-12 conditioned BCG infected CFP10-DCs would mediate killing of *M. tb* H37Rv inside macrophages. As shown in Figure 9, IFN-γ and IL-12 conditioned BCG infected CFP10-DCs significantly killed *M. tb* H37Rv inside macrophages. In fact, the bacterial loads were 3 fold lower than IFN-γ treated macrophages. Similar results were obtained when CFP10-DCs were transformed with retroviruses expressing IFN-γ or IL-12. Recombinant retrovirus transformed CFP10-DCs induced 3-fold lower CFU than IFN-γ treated macrophages, indicating a better effect of conditioned DCs in mediating killing of *M. tb* inside macrophages than that observed with IFN-γ.

**Figure 10:**
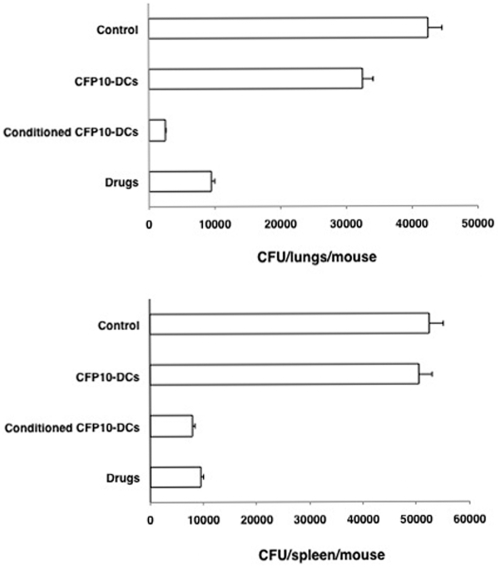
IFN-γ or IL-12 conditioned CFP10-DCs mediate clearance of *M. tb* infection in vivo in mice. Groups of mice were infected with 1×10^6^ *M. tb* H37Ra intravenously via the tail vein. Seven days post-infection, 10×10^6^ unconditioned CFP10-DCs or CFP10-DCs treated with both IFN-γ and IL-12 together (2 ng/ml each) for 2h (conditioned CFP10-DCs) were injected into the tail vein of mice. A repeat injection with the same number of DCs was given 7 days following the first transfer. ‘Control’ represents mice infected with *M. tb* H37Ra followed by intravenous injection of PBS. Seven days following the 2^nd^ transfer, mice were sacrificed and lung and spleen homogenates were plated onto 7H11 agar plates in serial dilutions for CFU monitoring. In parallel, infected mice were injected with anti-TB drugs as given in *Materials and Methods*. Data are the mean±s.d. of three experiments. For lungs, P<0.04 (Control vs Conditioned CFP10-DCs), P<0.03 (CFP10-DCs vs conditioned CFP10-DCs). For spleen, P<0.02 (Control vs Conditioned CFP10-DCs), P<0.02 (CFP10-DCs vs conditioned CFP10-DCs).

We further extended the above observations to in vivo infection in mice. Mice were first infected with *M. tb* H37Ra followed by adoptive transfer of IFN-γ and IL-12 conditioned CFP10-DCs. CFU in lungs and spleen were recorded at the end of the experiment. As shown in Figure 10, similar to RANTES and IP-10 conditioned CFP10-DCs, IFN-γ and IL-12 conditioned CFP10-DCs mediated effective clearance of an established *M. tb* infection from both lungs and spleen. In fact, the extent of clearance was similar to that observed following chemotherapy with drugs, once again indicating similar kinetics of clearance. These results indicate the potential of IFN-γ and IL-12 conditioned antigen specific DCs to clear an established *M. tb* infection.

## Discussion

A number of studies document the use of *M. tb* antigens in diagnostics and as potential ‘vaccine candidates’ [23]. We have been characterizing the interactions of some of these antigens with DCs and have observed that many antigens induce the differentiation and maturation of DCs [7], [8]. These DCs, however, induce suppressor responses [9]. Since chemokines regulate the quality of immune responses to bacterial infections, in the present investigation, we characterized the roles played by two pro-inflammatory response promoting chemokines RANTES and IP-10 during interactions of CFP10-DCs with mycobacteria.

A number of studies document changes in chemokine profiles following infection by *M. tb* [24]–[26]. *M. tb* infection increases expression of RANTES and its receptor CCR5 [27]. Induction of RANTES was also observed following stimulation of mononuclear cells with *M. tb* HSP70 [28]. It is now well established that distinct chemokines induce and qualitatively regulate T helper functions [29]. For example, RANTES is known to induce Th1 responses [30]. BCG vaccination enhances RANTES expression and Th1 responses in guinea pig models of tuberculosis [31]. Similarly, expression of IP-10 is associated with pro-inflammatory responses [32]. In contrast, many chemokines including eotaxin, MDC, TARC and I-309, induce suppressor responses [33]. Further, the chemokine expression patterns of DCs have also been extensively investigated and data suggest that DCs are responsive to various chemokines that are both stage and activation dependent [34].

In the light of the above, it was of interest to investigate the expression profiles of RANTES and IP-10 in CFP10-DCs and their role in influencing immune responses during mycobacterial infection in vitro and in vivo. Our results indicated that mycobacteria infected CFP10-DCs downregulate the expression of RANTES and IP-10 and induce suppressor responses to mycobacteria. Conditioning CFP10-DCs with RANTES or IP-10 induced pro-inflammatory immune responses to BCG. On the other hand neutralizing RANTES or IP-10 in GM-CSF-DCs abolished inflammatory responses, thereby indicating a positive role of RANTES and IP-10 in influencing the quality of immune responses from DCs during *M. tb* infection. Conditioning CFP10-DCs with RANTES possibly result in increased avidity onto CCR5 on these DCs following their interactions with BCG thereby resulting in increased CCR5 mediated effects. Similarly, conditioning with IP-10 may provide increased ligand density to CXCR3 with similar effects.

To extend the observations on RANTES and IP-10 to other chemokines, we analyzed the expression levels of a range of chemokines and their receptors in GM-CSF-DCs and CFP10-DCs following infection with BCG using pathway specific microarray. In concurrence with the results obtained on RANTES and IP-10, the expression levels of many chemokines and their receptors known to mediate pro-inflammatory responses were poorly expressed in BCG infected CFP10-DCs when compared with BCG infected GM-CSF-DCs. Prominent among them were CCR5 and CCR1 that attract RANTES [6]. CCR7 that is important for homing of DCs to T cell rich areas was also expressed at low levels. This could result in reduced priming of antigen specific T cells. Further, keeping the chemokine receptor redundancy in mind (there are about 18–20 receptors for over 50 chemokines) these results suggested that downregulation of receptor levels could also result in reduced responsiveness to many other chemokines resulting in decreased avidity of interactions. In contrast, compared to GM-CSF-DCs, mycobacteria infected CFP10-DCs showed higher expression of chemokines-TARC and eotaxin-that promote suppressor responses. The expression of type 1 IFNs that suppress pro-inflammatory responses during DC:T cell interactions [20], [21], was highly upregulated in CFP10-DCs both prior to and following infection with BCG.

Next, to give functional relevance to the above observations we tested whether increased expression of RANTES or IP-10 in DCs would contribute towards enhanced clearance of mycobacteria from infected macrophages. An important function of DCs is to prime T cells [5] that then mediate effector functions, e.g. activation of infected macrophages in the case of *M. tb* infection [22]. Our results showed that T cells activated by RANTES or IP-10 conditioned CFP10-DCs, harbor a Th1 phenotype and mediate enhanced killing of BCG from infected macrophages when compared with untreated CFP10-DCs. In fact, T cells from unconditioned CFP10-DCs enhanced CFU loads in macrophages perhaps as a result of enhanced IL-10 production from T cells, thereby reiterating our earlier observations for a negative role of CFP10-DCs in mediating protective responses to mycobacteria [35]. In addition, we also showed that T cells recruited by RANTES and IP-10 conditioned CFP10-DCs mediate effective killing of mycobacteria inside macrophages. These results have important bearings on the ability of chemokines RANTES and IP-10 in conditioning DCs to prime effector T cell responses both at the level of recruitment and subsequently at the level of effector functions leading to elimination of mycobacteria from macrophages.

To test proof of principle, we then tested the ability of RANTES and IP-10 conditioned CFP10-DCs to clear an established infection in mice. We showed that while unconditioned CFP10-DCs were ineffective in clearing *M. tb* infection, RANTES and IP-10 conditioned CFP10-DCs mediated effective clearance of *M. tb* infection in mice that was as effective as treatment with drugs, thus giving physiological relevance to the data obtained thus far.

An important aspect that regulates chemokine expression and effector functions is the cross-regulation by cytokines. Cytokines such as IFN-γ directly induce the expression of chemokines [36]. We therefore explored this aspect in CFP10-DCs and its effects on clearing *M. tb* infection. We showed that IFN-γ treatment restored the downregulation of RANTES and IP-10 levels from CFP10-DCs. We had earlier shown that mycobacterial stimulation of CFP10-DCs downregulates the expression of IL-12p40 and IFN-γ such that conditioning CFP10-DCs with these cytokines induced Th1 responses [9]. We extended those observations to the present experimental set up and showed that T cells activated from IL-12p40 and IFN-γ conditioned CFP10-DCs indeed mediated killing of virulent *M. tb* H37Rv inside macrophages. Further, IL-12 conditioned CFP10-DCs resulted in clearance of in vivo *M. tb* infection that was 3 fold better than that obtained following treatment with drugs.

It has been reported that ligation of IL-12 on the IL-12 receptor on monocyte derived DCs results in the upregulation of IL-12p40 [37]. Further, IL-12 treatment of DCs also results in the activation of a number of signaling molecules such as Janus kinase 2, Tyk2 kinases and the recruitment of several tyrosine phosphorylated proteins to IL-12Rβ, together with the activation of Stat3 and Stat4 transcription factors. In addition, IL-12 conditioning of DCs also increases the expression of many pro-inflammatory cytokines such as TNF-α, IL-1β, IL-6 and IFN-γ. Grohmann et al [38] also reported that IL-12 directly acts on DCs to activate the transcription factor NF-κB resulting in increased secretion of IL-12 from DCs. This indicates that IL-12 acts on the IL-12 receptor to program DCs for mounting pro-inflammatory responses. In the light of the above our data indicate that IL-12 conditioning of CFP10-DCs would result in the restoration of IL-12p40 levels that are downregulated following mycobacterial stimulation; further suggesting that one of the mechanisms of protection offered by IL-12 conditioned CFP10-DCs, when they come in contact with *M. tb* in infected mice, could be the activation of signaling molecules and increased expression of pro-inflammatory cytokines that would collectively program CFP10-DCs to induce protective responses. This would result in better clearance of *M. tb* from cytokine conditioned CFP10-DCs as opposed to poor clearance from unconditioned CFP10-DCs.

The above results emphasize the importance of pro-inflammatory Th1 responses from antigen specific DCs during *M. tb* infection. The characterizing of Th1/Th2 profiles during various stages of tuberculosis infection has exemplified the importance of the critical balance this ratio exerts in regulating the onset, progression and clearing of infection. A number of studies in humans indicate a prevalence of Th2 inducing cytokines at sites of infection with the progression of disease [40]–[42]. In addition, chemotherapy induces a change from a Th0 to Th1 cytokine profile. [43], [44]. It was also observed that when a Th2 response is superimposed upon a pre-existing Th1 response, the resulting cell-mediated inflammatory site becomes sensitive to cytokine-mediated damage. This indicated a role for a Th2 component in the immune response of tuberculosis patients. Therefore, it has been proposed that one should aim to switch off this Th2 component [45]–[47].

The results presented by us in this study in a sense have achieved a superimposition of pro-inflammatory Th1 responses over suppressor Th2/Th0 responses that eventually mediated clearing of the infection. This was made possible by the use of chemokine (RANTES and IP-10) or by cytokine (IFN-γ and IL-12) conditioned antigen specific CFP10-DCs, that induced pro-inflammatory responses in a Th2/Th0 environment culminating in effective clearance of an established infection.

DCs have shown lot of promise in providing protective immunity to many disease conditions including mycobacterial infections [48]. Antigen pulsed DCs have been used to monitor immune responses during *M. tb* infection [49]. In a monkey model of tuberculosis, Marino et al. [50] demonstrated the importance of early sensing of *M. tb* infection by DCs, their migration to the lymph nodes and T cell trafficking. It was demonstrated that for effective protection, an early activation and migration of DCs to draining lymph nodes is required. This would lead to stimulation of antigen specific T cells and delays in any of the above could significantly alter the outcome of mycobacterial infections. Further, it was emphasized that new and better vaccines should elicit a fast DC turnover at sites of infection together with strong DC activation ensuring maximum antigen presentation to T cells that result in the production of key cytokines required for inducing protective immunity.

These results emphasize the role of DCs in mediating protective responses to *M. tb*. However, studies on the role of DCs offering protection in the context of an established *M. tb* infection that is reminiscent with the physiological scenario have not been conducted. To this end, our results indicate the potential of antigen specific conditioned DCs to induce protective immune responses to an established infection and add support to the observations by Marino et al [50] who emphasize on the roles of chemokines and cytokines and T cell activation for achieving the same.

**Figure 11:**
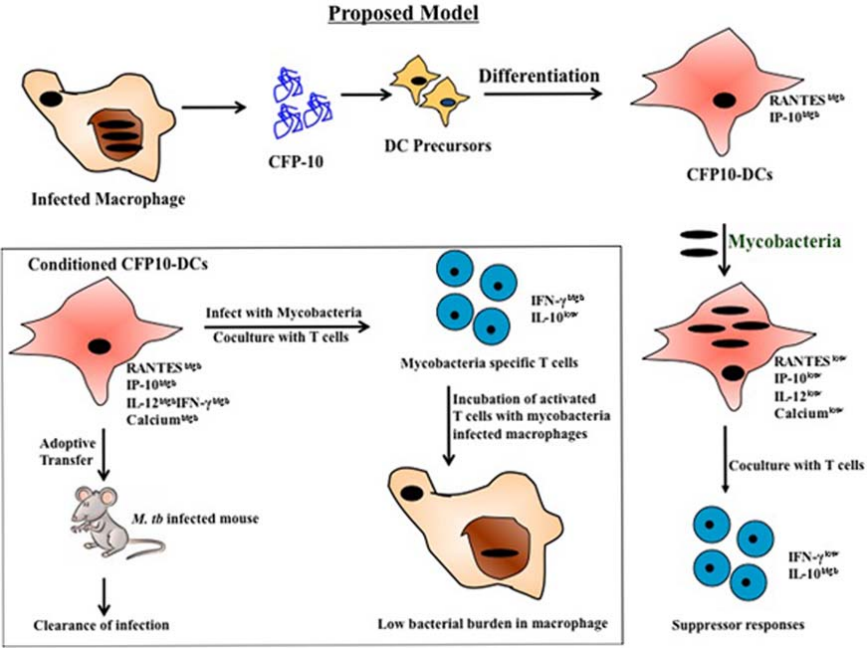
Conditioning CFP10-DCs with chemokines or cytokines induces protective responses. Following infection by *M. tb* and their sequestration inside alveolar macrophages, antigens such as CFP-10 are secreted. CFP-10 induces the differentiation of DCs. CFP10-DCs secrete high levels of RANTES and IP-10. Following their interactions with mycobacteria CFP10-DCs reverse their phenotype and secrete low levels of RANTES, IP-10 and IL-12p40 and display poor calcium influx. This leads to increased survival of mycobacteria and the induction of suppressor T cell responses. Box, conditioning CFP10-DCs with either RANTES and IP-10 or IL-12 and IFN-γ induced pro-inflammatory T cell responses and T cells activated by these DCs mediate killing of *M. tb* inside infected macrophages. In addition, adoptive transfer of chemokine and cytokine conditioned CFP10-DCs into mice harboring an active *M. tb* infection results in clearance of infection that is commensurate with drug treatment.

In summary, our results indicate that antigens such as CFP-10 are secreted by *M. tb* and induce the differentiation of DCs at sites of infection. Following interactions with mycobacteria, CFP10-DCs downmodulate the levels of pro-inflammatory chemokines and cytokines and display changes in the levels of key second messengers such as calcium [Figure 11]. This not only leads to the development of suppressor T cell responses to mycobacteria, but also results in these DCs serving as a harbor for mycobacterial survival and multiplication [17], [35]. Conditioning CFP10-DCs with pro-inflammatory chemokines or cytokines reverses these changes [Figure 11, box]. Conditioned DCs activate T cells that mediate effective killing of mycobacteria from macrophages. Importantly, in vivo transfer of conditioned CFP10-DCs offers protective immunity to an established *M. tb* infection in mice thus offering alternative strategies for vaccine design and the treatment of tuberculosis. The fact that the extent of protection was similar to that obtained with drug treatment indicates the potential of antigen specific conditioned DCs along with drug treatment to get effective clearance of established *M. tb* infection.

## Materials and Methods

### Animals

Female BALB/c mice 4–6 wk of age kept in pathogen free environment were used following approval from the Institutional Animal Ethics Committee.

### Materials

Recombinant mouse chemokines, cytokines, neutralizing antibodies and ELISA kits were from R&D Systems. Antibodies for FACS were from BD Pharmingen. Pathway specific chemokine and chemokine receptor arrays (catalog # MM 005) along with AmpLabeling kits (cat # L03) were from SuperArray. General reagents were from Sigma.

### Expression and purification of CFP-10

Endotoxin free CFP-10 was recombinantly expressed and purified from *E. coli* as described earlier [7]. The endotoxin levels was <0.3 EU/mg protein.

### Generation of DCs

DCs were differentiated from lymphocyte^−^ and I-A^−^ bone marrow precursors with either 15 ng/ml GM-CSF or 20 μg/ml CFP-10. This method gives a homogenous population that is 99% DCs with negligible contaminating monocytes/macrophages [7], [17].

### Infection of DCs with M. bovis BCG

*M. bovis* BCG or *M. tb* H37Rv were grown in Middlebrook 7H9 liquid medium supplemented with OADC (oleic acid/albumin/dextrose/catalase) with 0.05% Tween 80. DCs were infected with BCG at a Multiplicity of Infection (MOI) 1 and chemokines and cytokines were measured by ELISA. For some experiments macrophages were infected with either 1 MOI BCG or 1 MOI *M. tb* H37Rv for indicated times.

### In vivo primary responses with BCG

DCs were infected with 1 MOI BCG for 24h as described above. Following extensive washes to remove extracellular bacteria, infected or control uninfected DCs (5×10^6^/mouse) were injected into naïve mice subcutaneously at base of tail. Seven days later the inguinal lymph nodes were excised and 5×10^6^ cells/ml were cultured in RPMI 1640 medium with 10% FCS for 48h and cytokines in culture supernatants were scored by ELISA.

### Electrophoretic Mobility Shift Assay (EMSA)

20 μg of nuclear extract prepared as described earlier [7] from DCs stimulated with 1 MOI of BCG for various times was incubated with either 250 fmol of ^32^P-end-labeled double stranded oligonucleotide corresponding to either -67 to -99 in the RANTES promoter [13] or ^32^P-end-labeled double stranded oligonucleotide corresponding to -99 to -118 in the IP-10 promoter [14] for 15 min at 37^o^C. The incubation mixture included 3 μg of double stranded poly dI-dC in a binding buffer (25 mM Hepes pH 7.9, 0.5 mM EDTA, 0.5 mM DTT, 1% Nonidet P-40, 5% glycerol, 50 mM NaCl). The DNA-protein complex was separated from free oligonucleotide on a 5% native polyacrylamide gel using buffer containing 50 mM Tris, 200 mM glycine (pH 8.5) and 1 mM EDTA. Blots were visualized using a phosphoimager (Molecular Dynamics, Typhoon (210) and bands were quantified using the inbuilt ‘IMAGEQUANT TL’ software.

### Estimation of intracellular calcium levels

Intracellular calcium levels were monitored as described recently [17]. Briefly, either 2×10^7^/ml GM-CSF-DCs or CFP10-DCs were loaded with 1 μM FLUO-3-AM for 45 min at 37°C in culture medium. Labeled cells were stimulated with 1 MOI BCG and real time increase in intracellular calcium levels were monitored immediately over a period of 5 min by flow cytometry using FACSCalibur (Beckton & Dickinson) and the data were analyzed employing the CellQuest Pro software. For some experiments CFP10-DCs were incubated with 5 ng/ml recombinant RANTES or IP-10 for 12h, while GM-CSF-DCs were incubated with 10 μg/ml neutralizing monoclonal antibody to RANTES or IP-10 for 2h, prior to stimulation with BCG.

### Microarray experiments and analyses

Total RNA was enriched from CFP10-DCs or GM-CSF-DCs infected for 24h with 1 MOI BCG. GEArray Q Series mouse chemokine and receptor gene array from SuperArray was employed along with the Amplabelling kit. These are cDNA arrays containing a total of 67 chemokines and chemokine receptor genes along with house keeping genes. All steps were carried out strictly following the manufacturer's instructions. Blots were visualized using a phosphoimager (Molecular Dynamics, Typhoon 210) and bands quantified using the inbuilt ‘IMAGEQUANT TL’ software.

### Immunization of mice with BCG for T cell enrichment

Mice were immunized subcutaneously at base of tail with 1×10^6^ BCG/mouse. Seven days later T cells from the inguinal lymph nodes were enriched as described before [7]. Briefly, B cells and MHC class II^+^ cells were depleted by two rounds of incubation with CD19^+^ and I-A^+^ MACS beads. The purity of the enriched T cells was 98% as ascertained by FACS. The percentage of I-A^+^ cells was <0.05%.

### Migration of antigen specific T cells to chemokine gradients

CFP10-DCs or GM-CSF-DCs were infected for 24h with 1 MOI BCG. Culture supernatants were filtered through 0.2 μM membrane and 0.6 ml placed in the lower chamber of a Transwell apparatus (Corning, USA catalog # 3421) fitted with a 6 mm diameter membrane having a 5 micron pore size. In the upper chamber, 0.1×10^6^ enriched T cells from BCG immunized mice in 0.1 ml culture medium were added. For some groups CFP10-DCs were conditioned with 5 ng/ml recombinant RANTES or IP-10 for 12h prior to infection with BCG. Following 2h of incubation, the cells migrated into the lower chamber were harvested and counted. T cells were then incubated with BCG infected macrophages as described below.

### In vitro clearance of mycobacteria from macrophages by DC-activated T cells

CFP10-DCs were conditioned with either 25 ng/ml RANTES or IP-10 for 12h prior to infection with BCG. Alternatively, CFP10-DCs were conditioned with 2 ng/ml IFN-γ or IL-12p70 for 2h or transformed with retrovirus expressing IFN-γ or IL-12p40 for 12h prior to infection with BCG. Conditioned BCG infected CFP10-DCs were co-cultured for 48h with BCG specific T cells enriched from immunized mice. From this DC:T cell co-culture, DCs were depleted by MACS and T cells were cultured for 48h with peritoneal macrophages that were earlier infected for 24h with either 1 MOI BCG or 1 MOI *M. tb* H37Rv. For some experiments, T cells recruited by RANTES or IP-10 conditioned BCG infected CFP10-DCs were cultured with BCG infected macrophages for 48h. Cells were lysed and lysate was plated onto 7H11 agar plates in serial dilutions. Colony Forming Units (CFU) were determined 2–3 week later.

### Infection of mice with M. tb and adoptive transfer of DCs

Groups of naïve mice (5 mice/group) were infected with 1×10^6^ *M. tb* H37Ra via the tail vein. 24h later one group of mice were sacrificed and lung homogenates were plated onto 7H11 agar plates for confirming establishment of infection. Seven days post infection, 10×10^6^ uninfected DCs were injected into the tail vein of mice. A repeat injection with the same number of uninfected DCs was carried out 7 days following the first transfer. Seven days following the 2^nd^ transfer, mice were sacrificed and lung and spleen cells were enriched using a homogenizer. An aliquot of the homogenate was lysed and plated onto 7H11 agar plates in serial dilutions for CFU monitoring.

### Retroviral transformation of DCs

Splenocytes from naïve mice were stimulated with concanavalin A. mRNA was enriched and used to generate full-length cDNAs for IL-12p40 and IFN-γ by RT-PCR. The cDNAs were then separately cloned into pLNCX2 retroviral vector (CLONTECH, San Diego). The recipient cell line PT67 was then transfected with the plasmid to generate replication incompetent recombinant retroviruses expressing IL-12p40 or IFN-γ. CFP10-DCs were transformed with IL-12p40 or IFN-γ expressing retroviruses for 12h and expression of cytokines (Figure S7) was monitored by RT-PCR.

### Treatment of M. tb infected mice with Drugs

Following 7 days of infection mice were treated with oral administration of Isoniazid 25 mg/Kg-body-weight, Ethambutol 100 mg/Kg-body-weight and Rifampicin 20 mg/Kg-body-weight essentially following Nikonenko et al. [51]. A repeat dose was given 7 days after and on every alternate day till mice were sacrificed.

### Statistical Analysis

Student's ‘t test was performed to test the statistical significance of the differences in means of various groups. In all experiments P values<0.05 were considered as significant.

## Supporting Information

Figure S1 CFP10-DCs and GM-CSF-DCs show similar maturation and uptake of BCG. For Panel A, BCG infected CFP10-DCs or GM-CSF-DCs were stained for surface expression of MHC class II (purple), MHC class I (orange), CD54 (green), CD40 (blue) CD86 (black) and CD80 (red) and analyzed by FACS. Data are expressed as levels of mean fluorescence intensity (MFI) relative to uninfected controls. Panel B shows Dil C 18 labeled BCG infected GM-CSF-DCs (left panel) and CFP10-DCs (right panel). The thick lines and thin lines in both the panels represent infected and uninfected DCs, respectively. Data from one of four independent experiments are shown.
(0.16 MB TIF)

Figure S2 LPS induces activation of CFP10-DCs and GM-CSF-DCs. CFP10-DCs or GM-CSF-DCs were either stimulated with 0.5 μg/ml LPS or infected with 1 MOI BCG for 24h. RANTES and IP-10 levels in supernatants were measured. Data from one of two independent experiments are shown.
(0.08 MB TIF)

Figure S3 MTSA and ESAT6 form a tight 1:1 complex. Far UV Circular Dichroism spectra of MTSA (profile a), ESAT6 (profile b) and MTSA:ESAT6 heterodimer (profile c). The MTSA and ESAT6 dimer was generated following Renshaw et al. 2002, J. Biol. Chem. 277: 21598-21603. The spectrum was generated for 10 μM MTSA or ESAT6 or MTSA:ESAT6 dimer in 25 mM NaH2PO4 buffer at pH 6.5 using a JASCO spectrometer model J810 as described by Renshaw et al. 2002. The spectra were recorded at 250C in a 2 mm path length cell from 190 to 250 nm at a scan speed of 100 nm/min, with each spectrum representing an average of 4 accumulations. The spectra for all three proteins match those reported by Renshaw et al. 2002. While MTSA displays a unstructured random coiled polypeptide, ESAT6 and the MTSA:ESAT6 dimer display profiles typical of proteins with a helical structure
(0.07 MB TIF)

Figure S4 DCs differentiated with CFP10:ESAT5 dimer downregulate RANTES and IP-10 expression upon BCG infection. DCs were differentiated with CFP-10 and ESAT-6 dimer (Dimer-DCs) and subsequently infected with 1 MOI BCG for 24h. Culture supernatants were screened for the levels of RANTES or IP-10 by ELISA.
(0.07 MB TIF)

Figure S5 CFP10-DCs show reduced recruitment of NF-κB to RANTES and IP-10 promoter. Either CFP10-DCs or GM-CSF-DCs were infected with 1 MOI BCG for indicated times. 20 μg of nuclear extracts were incubated with 32P-end-labeled oligonucleotide from the RANTES promoter (Panel A) or the IP-10 promoter (Panel B) and EMSA was performed. FP depicts free probe. Panel C shows a representative of cold competition with wild-type (wt) and mutant (mut) consensus NF-κB probe in EMSA with the IP-10 promoter. Numbers below EMSA represent relative intensities of the bands.
(0.18 MB TIF)

Figure S6 CFP10-DCs show reduced recruitment of CD69+ CD4+ T cells. GM-CSF-DCs (Upper panel) or CFP10-DCs (Lower panel) were uninfected (Left panel) or infected (Right panel) with BCG for 24h. Culture supernatants (0.6 ml) were placed in the lower chamber of a Transwell apparatus fitted with a 6 mm diameter membrane having a 5.0 micron pore size. In the upper chamber 0.1×106 BCG specific enriched T cells in (0.1 ml) were added. Following 2h of incubation the cells migrated into the lower chamber were stained for the surface expression of CD4 and CD69. One of three independent experiments is shown.
(0.07 MB TIF)

Figure S7 DCs transformed with retrovirus encoding IFN-{lower case gamma or IL-12p40 express mRNA of the transformed cytokines. A, CFP10-DCs were infected with either a control retrovirus (V) or retrovirus expressing IFN-{lower case gamma (IFN) or IL-12p40 (IL12) for 12h. Total RNA was enriched from cells and subjected to RT-PCR for full-length expression of IFN-γ or IL-12p40 mRNA.
(0.07 MB TIF)
