# Supplementary figures and images for: Protective Immunity to Mycobacterium tuberculosis Infection by Chemokine and Cytokine Conditioned CFP-10 Differentiated Dendritic Cells
Source: PLoS One. 2008 Aug 6;3(8):e2869. doi: 10.1371/journal.pone.0002869 (PMC2478708; doi:10.1371/journal.pone.0002869)

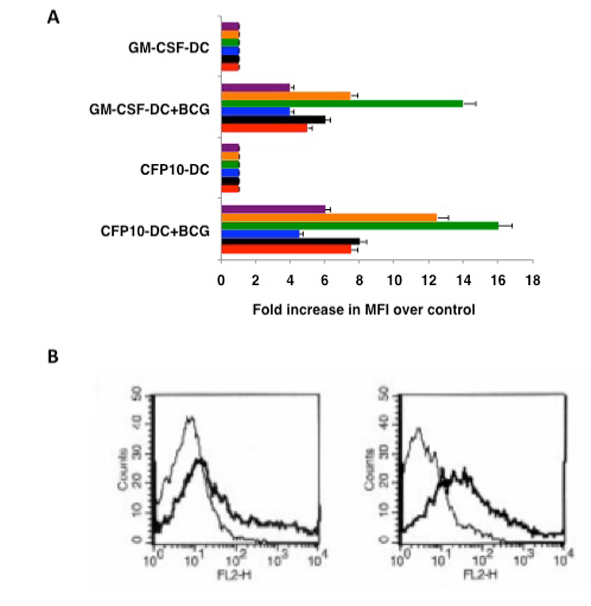

Supplement: Figure S1 — CFP10-DCs and GM-CSF-DCs show similar maturation and uptake of BCG. For Panel A, BCG infected CFP10-DCs or GM-CSF-DCs were stained for surface expression of MHC class II (purple), MHC class I (orange), CD54 (green), CD40 (blue) CD86 (black) and CD80 (red) and analyzed by FACS. Data are expressed as levels of mean fluorescence intensity (MFI) relative to uninfected controls. Panel B shows Dil C 18 labeled BCG infected GM-CSF-DCs (left panel) and CFP10-DCs (right panel). The thick lines and thin lines in both the panels represent infected and uninfected DCs, respectively. Data from one of four independent experiments are shown. (0.16 MB TIF) [file pone.0002869.s001.tif]

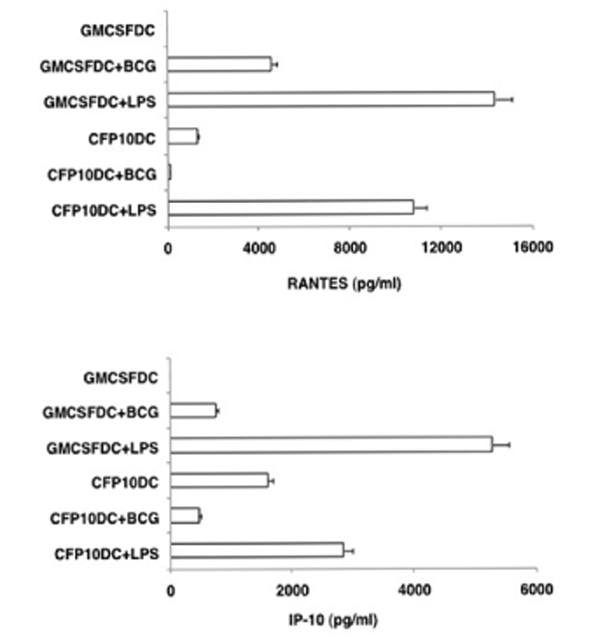

Supplement: Figure S2 — LPS induces activation of CFP10-DCs and GM-CSF-DCs. CFP10-DCs or GM-CSF-DCs were either stimulated with 0.5 μg/ml LPS or infected with 1 MOI BCG for 24h. RANTES and IP-10 levels in supernatants were measured. Data from one of two independent experiments are shown. (0.08 MB TIF) [file pone.0002869.s002.tif]

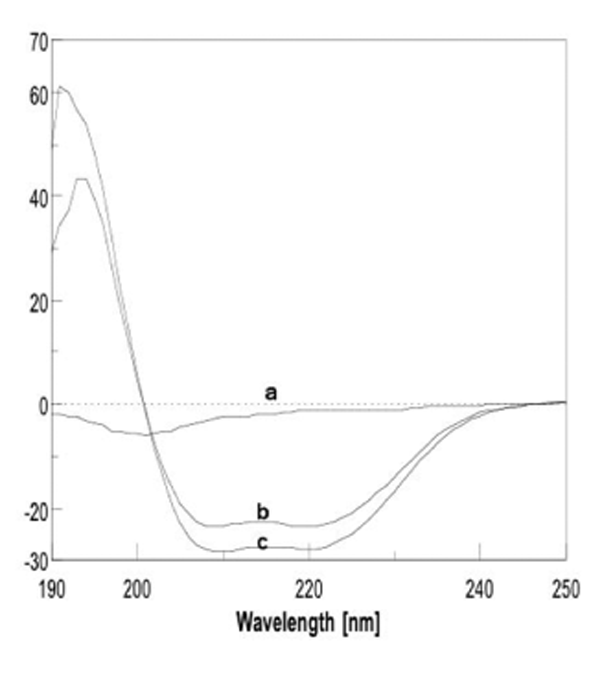

Supplement: Figure S3 — MTSA and ESAT6 form a tight 1:1 complex. Far UV Circular Dichroism spectra of MTSA (profile a), ESAT6 (profile b) and MTSA:ESAT6 heterodimer (profile c). The MTSA and ESAT6 dimer was generated following Renshaw et al. 2002, J. Biol. Chem. 277: 21598-21603. The spectrum was generated for 10 μM MTSA or ESAT6 or MTSA:ESAT6 dimer in 25 mM NaH2PO4 buffer at pH 6.5 using a JASCO spectrometer model J810 as described by Renshaw et al. 2002. The spectra were recorded at 250C in a 2 mm path length cell from 190 to 250 nm at a scan speed of 100 nm/min, with each spectrum representing an average of 4 accumulations. The spectra for all three proteins match those reported by Renshaw et al. 2002. While MTSA displays a unstructured random coiled polypeptide, ESAT6 and the MTSA:ESAT6 dimer display profiles typical of proteins with a helical structure (0.07 MB TIF) [file pone.0002869.s003.tif]

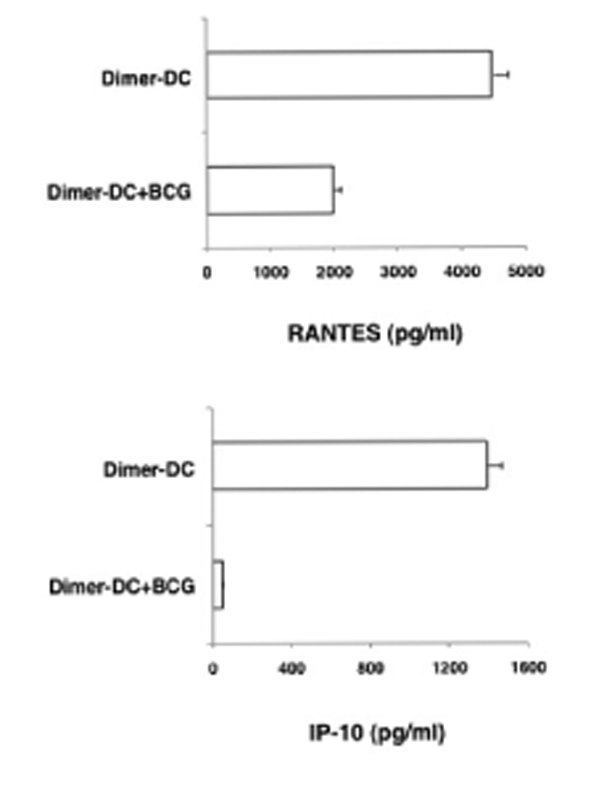

Supplement: Figure S4 — DCs differentiated with CFP10:ESAT5 dimer downregulate RANTES and IP-10 expression upon BCG infection. DCs were differentiated with CFP-10 and ESAT-6 dimer (Dimer-DCs) and subsequently infected with 1 MOI BCG for 24h. Culture supernatants were screened for the levels of RANTES or IP-10 by ELISA. (0.07 MB TIF) [file pone.0002869.s004.tif]

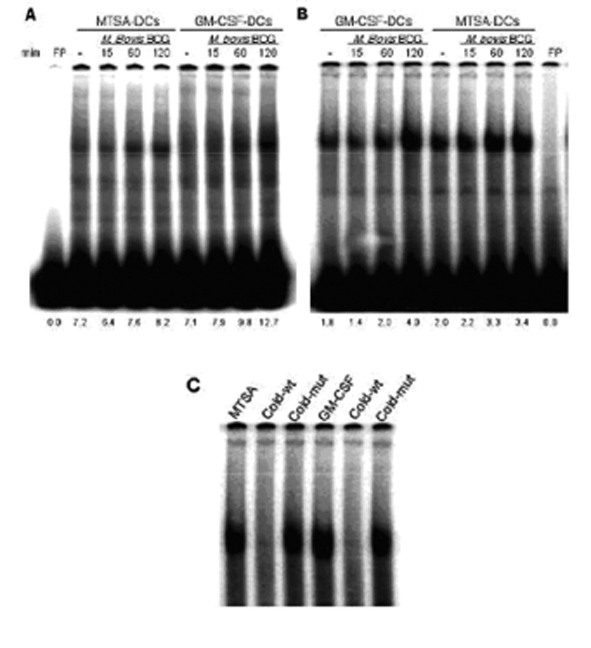

Supplement: Figure S5 — CFP10-DCs show reduced recruitment of NF-κB to RANTES and IP-10 promoter. Either CFP10-DCs or GM-CSF-DCs were infected with 1 MOI BCG for indicated times. 20 μg of nuclear extracts were incubated with 32P-end-labeled oligonucleotide from the RANTES promoter (Panel A) or the IP-10 promoter (Panel B) and EMSA was performed. FP depicts free probe. Panel C shows a representative of cold competition with wild-type (wt) and mutant (mut) consensus NF-κB probe in EMSA with the IP-10 promoter. Numbers below EMSA represent relative intensities of the bands. (0.18 MB TIF) [file pone.0002869.s005.tif]

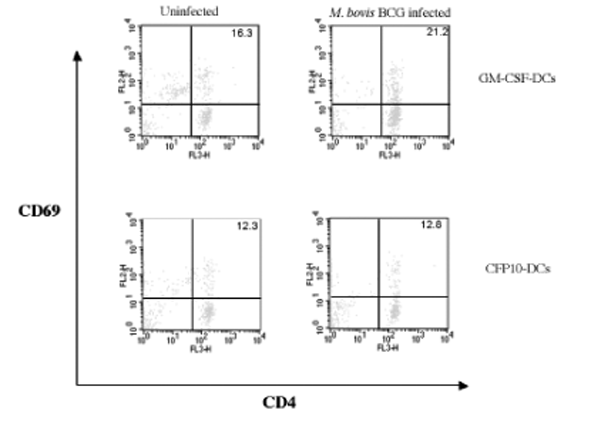

Supplement: Figure S6 — CFP10-DCs show reduced recruitment of CD69+ CD4+ T cells. GM-CSF-DCs (Upper panel) or CFP10-DCs (Lower panel) were uninfected (Left panel) or infected (Right panel) with BCG for 24h. Culture supernatants (0.6 ml) were placed in the lower chamber of a Transwell apparatus fitted with a 6 mm diameter membrane having a 5.0 micron pore size. In the upper chamber 0.1×10^6^ BCG specific enriched T cells in (0.1 ml) were added. Following 2h of incubation the cells migrated into the lower chamber were stained for the surface expression of CD4 and CD69. One of three independent experiments is shown. (0.07 MB TIF) [file pone.0002869.s006.tif]

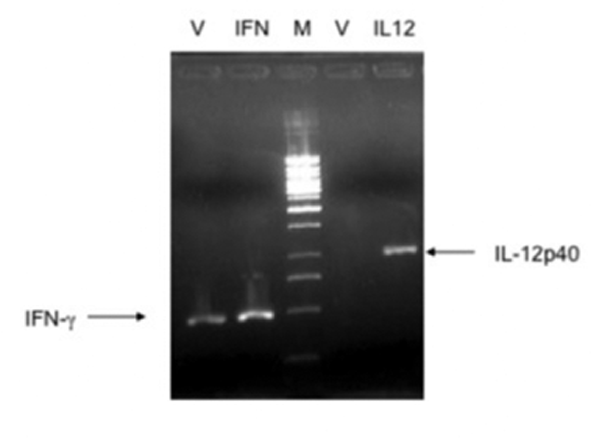

Supplement: Figure S7 — DCs transformed with retrovirus encoding IFN-{lower case gamma or IL-12p40 express mRNA of the transformed cytokines. A, CFP10-DCs were infected with either a control retrovirus (V) or retrovirus expressing IFN-{lower case gamma (IFN) or IL-12p40 (IL12) for 12h. Total RNA was enriched from cells and subjected to RT-PCR for full-length expression of IFN-γ or IL-12p40 mRNA. (0.07 MB TIF) [file pone.0002869.s007.tif]
